# Supplementary material for: Phlpp1 is induced by estrogen in osteoclasts and its loss in Ctsk-expressing cells does not protect against ovariectomy-induced bone loss
Source: PLoS One. 2021 Jun 18;16(6):e0251732. doi: 10.1371/journal.pone.0251732 (PMC8213150; doi:10.1371/journal.pone.0251732)
Supplement: Attachment — Submitted filename: Response to Reviewers.docx [file pone.0251732.s002.docx]

**Reviewer #1:** In the manuscript “Phlpp1 is induced by Estrogen in osteoclasts and its loss in Ctsk-Expressing Cells Does Not Protect Against Ovariectomy-Induced Bone Loss” Hassan MK et al have described the influence of Phlpp1 in osteoclast activity in overiectomy mouse model in presence or absence of estrogen. The work might have a good contribution in osteoclast biology. However, the following issues need to be addressed for its improvement

1. Authors might explain about the “Ctsk-Cre expressing cells”, and full form of CTSK

*We delineated the Ctsk abbreviation within the manuscript; Ctsk-Cre expressing cells is used because the Ctsk-Cre transgene is expressed by different cell types.*

2. Full form of IGFBP4

*We defined Igfbp4 within the manuscript.*

3. Author should explain the procedure of isolation of osteoclast progenitor cells and its purity.

*We added this detail to the methods section of our manuscript.*

4. In figure 5, authors may show representative photos of TRAP positive osteoclast cells.

*We added these images to Figure 5.*

5. In figure 1, it would be nice if authors can show few photos of scanned via micro-CT

*Unfortunately, we do not have current access to some of the scans needed to generate reconstructions.*

**Reviewer #2:** In this manuscript, Hanson et al. document the effect of Ctsk-conditional Phlpp1 deletion on OVX-bone loss, and identify a potential mechanism by which estrogen regulates Phlpp1 and Igfbp4 expression. The underlying mechanism is very intriguing, however, there is a major issue with the overall premise. Specifically, since estrogen has effects on other bone cell lineages, including the osteoblast/osteocyte lineage, a loss of the increased bone phenotype with OVX does not specify a predominant role of Phlpp1 in resorption as compared to bone formation. In addition, there was no assessment of bone formation parameters in this model, beyond BV/TV, to support a lack of effect on coupling/bone formation. There are additional in vitro experiments/data that would strongly benefit this study.

Major comments:

1. As stated above, the rationale behind using OVX to test specificity for a resorptive vs formation effect of the Phlpp1 Ctsk-cre KO is flawed, as estrogen has effects on the osteoblast/osteocyte lineage, and OVX is known to impact osteoblast differentiation/bone formation.

*We revised our manuscript to reflect the Reviewer’s comments and added discussion of this limitation to our revised manuscript.*

2. The authors show there is no significant difference in bone phenotype between OVX WT and Ctsk Cre Phlpp1 KO animals. However, given that the sham phenotype is different, the authors should also assess percent change compared to sham. The fact that the animals have the same bone phenotype following OVX would suggest that the there was a more negative effect of OVX on bone mass in KO as compared to WT animals.

*We added these comparisons to Table 1 and discussed them within our revised manuscript.*

3. The authors showed that E2 increase Phlpp1 and decrease Igfbp4 protein levels and that Phlpp1 KO osteoclasts exhibited increased Igfbp4 expression. In order to fully understand this relationship and the impact on bone phenotype, the authors should do the same time course E2 treatment on WT and Phlpp1 KO osteoclasts.

*We added these data to Figure 4 and discussed them within our revised manuscript.*

4. In order to assess the impact of Ctsk Cre Phlpp1 KO or OVX on bone formation, the authors should assess dynamic bone formation rates and osteoblast number by histomorphometry. Current data only are reflective of the osteoclast data. This study would also benefit from serum markers of resorption and formation.

*We added these data to our manuscript.*

5. Given that osteoclasts could secrete IGFBP4, could this be a potential E2 induced coupling factor that increases bone formation in the sham mice?

*We added this provocative idea to our discussion.*
