# Supplementary material for: Phlpp1 is induced by estrogen in osteoclasts and its loss in Ctsk-expressing cells does not protect against ovariectomy-induced bone loss
Source: PLoS One. 2021 Jun 18;16(6):e0251732. doi: 10.1371/journal.pone.0251732 (PMC8213150; doi:10.1371/journal.pone.0251732)
Supplement: Attachment — Submitted filename: Reviewer comment-PLOS ONE.pdf [file pone.0251732.s001.pdf]

In the manuscript “**Phlpp1 is induced by Estrogen in osteoclasts and its loss in Ctsk-Expressing Cells Does Not Protect Against Ovariectomy-Induced Bone Loss”** Hassan MK et al have described the influence of Phlpp1 in osteoclast activity in overiectomy mouse model in presence or absence of estrogen. The work might have a good contribution in osteoclast biology. However, the following issues need to be addressed for its improvement

1. Authors might explain about the “Ctsk-Cre expressing cells”, and full form of CTSK
2. Full form of IGFBP4
3. Author should explain the procedure of isolation of osteoclast progenitor cells and its purity.
4. In figure 5, authors may show representative photos of TRAP positive osteoclast cells.
5. In figure 1, it would be nice if authors can show few photos of scanned via micro-CT
6. In figure 4, authors may provide the densitometry data of western blotting.
